# Assessing Knowledge, Attitude, and Practice of Twin Block Functional Therapy for Angle’s Class II Malocclusion Among Postgraduate Students of Pediatric Dentistry in India: A Cross-Sectional Survey

**DOI:** 10.7759/cureus.103770

**Published:** 2026-02-17

**Authors:** Abhisek Bhattacharjee, Richa Khanna, Rajeev K Singh, Ashmeetkaur Oberoi

**Affiliations:** 1 Pediatric and Preventive Dentistry, King George's Medical University, Lucknow, IND

**Keywords:** class ii malocclusion, interceptive orthodontics, orthodontic education, pediatric dentistry, twin block functional therapy

## Abstract

Context: Functional appliances such as Twin Block (TB) play a key role in managing Class II malocclusion during growth. However, limited data exist on pediatric dentists’ knowledge and clinical use of this therapy in India.

Aims: This study evaluated the knowledge, attitudes, and practices (KAP) of Indian postgraduate students in pediatric dentistry regarding Twin Block functional therapy (TBFT) for treating Class II malocclusion.

Settings and design: This was a questionnaire-based, cross-sectional study conducted among postgraduate students across India in March 2023.

Materials and methods: A cross-sectional survey was conducted using a structured questionnaire developed through standard methods. The questionnaire assessed knowledge, attitudes, and practical experiences related to Twin Block therapy.

Statistical analysis: A total of 135 postgraduate students from various dental institutions across India participated. Data analysis included descriptive statistics, Chi-square tests, and assessment of questionnaire reliability using Cohen’s kappa during pilot testing.

Results: The study revealed that knowledge of Twin Block therapy varied significantly with academic year, with second- and third-year students demonstrating increasingly better understanding. Only 11.8% (16 individuals) of respondents had adequate knowledge, while 62.2% (84 individuals) had average knowledge. Positive attitudes toward the therapy were noted among 78.5% (106 individuals) of participants, but only 47.4% (64 individuals) had treated Class II malocclusion cases. Challenges in patient compliance were reported by 88.2% (57 individuals) of respondents. A preference for part-time wear during meals was observed, differing from the recommended full-time use.

Conclusions: The findings highlight a gap between knowledge and practical application of Twin Block therapy among Indian pediatric dentistry postgraduates. There is a need for a more structured curriculum and increased clinical exposure to enhance competency in using functional appliances.

## Introduction

Interceptive and preventive orthodontic interventions offer early, cost-effective strategies to address developing malocclusions during the mixed dentition phase. Such early-phase treatments can reduce the need for comprehensive orthodontic care later in life [1] and are commonly employed to manage space loss, promote guided eruption, or influence skeletal growth [2,3]. Among these, functional appliances play a critical role in modulating jaw development. The Twin Block (TB) appliance, introduced by Clark [4], is among the most frequently used removable functional devices for managing Class II skeletal discrepancies by promoting mandibular advancement and improving facial aesthetics.

Evidence from randomized controlled trials and systematic reviews supports the effectiveness of the TB appliance in stimulating favorable skeletal changes, particularly when used during the growth spurt [1,4]. Given this, early intervention is essential. The American Association of Orthodontists recommends initial orthodontic assessment by age 7 [5], and the American Academy of Pediatric Dentistry (AAPD) emphasizes the pediatric dentist’s role in recognizing and managing developing occlusal issues [6].

International specialist training programs in pediatric dentistry, such as the UK’s General Dental Council (GDC) Specialty Curriculum, mandate formal training in interceptive and preventive orthodontic care, including management of malocclusion in growing children [7]. Although these documents do not specify Twin Block appliances by name, the broad curricular focus on early malocclusion management typically includes functional appliance modalities. In India, the Master of Dental Surgery (MDS) curriculum in Paediatric and Preventive Dentistry, regulated by the Dental Council of India (DCI), includes myofunctional therapy as a learning domain. However, specific competencies and clinical expectations related to TB therapy are not clearly defined. Institutional exposure, faculty expertise, and clinical postings may all influence the extent of student training in this area.

Despite the theoretical inclusion of functional appliance training, there is currently limited empirical evidence assessing postgraduate students’ preparedness to implement TB therapy in clinical practice. The present study seeks to address this gap. It does not aim to evaluate the curriculum itself or to make policy recommendations, but rather to explore current levels of knowledge, attitude, and clinical exposure among MDS students in pediatric dentistry.

This exploratory study aims to generate baseline data that could inform future, more comprehensive evaluations. The primary outcome of this study was the proportion of postgraduate students demonstrating adequate knowledge of Twin Block functional therapy (TBFT), as defined by a predefined scoring system. Functional appliances, including the Twin Block, have demonstrated measurable skeletal and soft tissue effects during adolescence, reinforcing their clinical significance in interceptive orthodontics [8].

Secondary outcomes included the proportion of students with prior clinical experience in managing Angle’s Class II malocclusion using functional appliances, as well as their attitudes toward TBFT and perceived barriers to its implementation. By identifying potential areas where theoretical knowledge may not translate into practical experience, it provides a snapshot of student readiness across a sample of institutions. These findings may help educators reflect on current teaching strategies and identify areas for improvement in clinical training related to TB therapy.

## Materials and methods

The questionnaire

The complete questionnaire used for data collection is provided in the Appendices. The questionnaire was developed following a standardized method proposed by Vaughn et al. [9], which included the following phases: (a) establishing a conceptual framework, (b) systematically creating an item pool, (c) refining the item pool through focus group discussions (FGD), (d) conducting validity testing, (e) performing translation and back-translation, (f) engaging in cognitive interviewing (CI), and (g) conducting reliability testing.

The initial conceptual framework was derived from core constructs outlined in Twin Block Functional Therapy by William J. Clark [4], including diagnostic parameters, appliance design, wear protocol, treatment goals, and relapse. To refine these into survey items, five FGDs were conducted with a purposive sample of postgraduate students (n=5), faculty members in pediatric dentistry (n=2), and senior residents (n=3) at our institution. Transcripts were thematically analyzed, reaching saturation at the fifth session. Key themes included diagnosis, appliance selection, wear-time protocols, patient compliance, relapse, and curricular gaps. Based on these, 30 draft items were created and mapped to relevant themes.

A panel of three content experts evaluated each item for relevance using a 4-point scale (1 = not relevant to 4 = highly relevant). Items with a mean relevance score below 3 or with significant thematic overlap were revised or excluded. This process led to a final questionnaire of 18 items. Responses were analyzed, leading to necessary adjustments in terminology and the number of items. Initially, a few knowledge-based questions were designed as single-best-answer items (e.g., multiple-choice questions (MCQs)), but were reformatted into 5-point Likert scale items during pilot testing to allow for a more nuanced capture of partial understanding and uncertainty. This change also aligned better with the knowledge, attitude, and practice (KAP) framework and improved the consistency of the response format.

The questionnaire’s validation involved a panel of eight experts who rated each item on a 4-point relevance scale (1 = not relevant, 2 = somewhat relevant, 3 = quite relevant, 4 = highly relevant). Ratings were used to calculate Lawshe’s content validity index (CVI) [10], which yielded a score of 0.91, indicating high content validity. Given that all study participants were presumed to be proficient in English, translation and back-translation of the questionnaire were considered unnecessary.

The pilot validation was conducted with a convenience sample of 15 postgraduate students, who evaluated item clarity, response time, and perceived redundancy. Feedback from this group was analyzed descriptively to identify items requiring linguistic simplification or structural modification before final administration.

Cognitive interviews (CI) with the same 15 participants were then conducted. Cognitive probes helped identify ease of comprehension and evaluate the thought process behind participants’ responses. Based on these observations, irrelevant items were removed, and questions were rephrased to reduce ambiguity.

The final questionnaire was developed as a Google Form (Google, Inc., Mountain View, CA) and distributed to postgraduate students from various dental colleges across India. The collected information included details on the type of workplace, such as whether it was government-funded or private. Additionally, data were gathered on the students’ year of residency (first, second, or third year) and the geographic region where their institutions were located (north, south, east, or west of India). The Google Form was configured to require a response for all items to minimize missing data. No adaptive branching logic was used; all participants viewed the same questionnaire. Duplicate submissions were restricted through mandatory institutional email sign-in.

Although the questionnaire did not explicitly label each item as assessing knowledge, attitude, or practice, the categorization was determined by expert consensus during the development phase. Items assessing factual understanding and principles of Twin Block therapy were grouped under “Knowledge,” those exploring beliefs, preferences, or perceptions were categorized as “Attitude,” and those concerning clinical usage and experience were designated as “Practice.” These internal groupings were used during analysis to stratify responses under knowledge, attitude, and practice domains, although the questionnaire presented them in a unified format.

Prospective respondents were contacted through multiple channels, including email links sent to postgraduate dental students at various teaching institutions, posts in closed dental groups on WhatsApp, and forwarded links shared through various messaging platforms among postgraduate students. To prevent multiple submissions by the same respondent, sign-in was required before completing the questionnaire.

Ethical considerations

This study was conducted as an anonymous, questionnaire-based survey involving postgraduate dental students. No patient data, identifiers, or sensitive personal information were collected. Participation was voluntary, and informed consent was obtained electronically prior to survey completion. The study adhered to ethical principles outlined in the Declaration of Helsinki for research involving human participants. Although email sign-in was required to avoid duplicate responses, these data were not used in the analysis and were kept separate from the dataset to maintain anonymity. All respondents were required to read the participant information sheet and electronically sign a consent form before participating in the survey. Although institutional ethics committee approval was not formally obtained at the time of study initiation, all procedures were designed to ensure participant confidentiality, voluntary participation, and data anonymity.

Statistics

Due to the study’s exploratory nature and lack of prior prevalence estimates, no formal sample size calculation was performed. However, efforts were made to ensure representation from all four regions of India and across public and private institutions. Participants were recruited through convenience sampling based on accessibility via institutional contacts, professional messaging groups, and peer referral networks.

To evaluate the stability of the questionnaire, a test-retest reliability check was conducted during the pilot phase with five randomly selected postgraduate students. The questionnaire was administered twice with a 14-day interval, and responses were analyzed using Cohen’s kappa.

Normality of the demographic data could not be ensured due to the use of convenience sampling. The data were coded and entered into Microsoft Excel (Microsoft Corp., Redmond, WA) and then imported into SPSS version 25 (IBM Corp., Armonk, NY) for statistical analysis. Coding here refers to the conversion of categorical responses (e.g., Likert scale options such as “Strongly Agree” to “Strongly Disagree”) into numerical values (e.g., 5 to 1) for statistical analysis. Each questionnaire item retained its original phrasing and was not renamed or modified in content; only the response categories were numerically coded to facilitate data entry, scoring, and group-wise comparisons.

The analysis involved using descriptive statistics to summarize the results and making comparisons among different groups. Categorical variables (e.g., year of study, institution type, and region) were presented as proportions and percentages, while ordinal and continuous variables were summarized as mean ± standard deviation (SD) where applicable. Quantitative data included responses to Likert scale items assessing attitudes and clinical practices, as well as ordinal ratings for perceived treatment success, patient compliance, and relapse rates. The Chi-square test was used for statistical comparisons of categorical and ordinal data across groups.

Knowledge items were scored on a 5-point Likert scale (1-5). For scoring purposes, responses were assigned numerical values from 1 (least correct/least appropriate response) to 5 (most correct/most appropriate response). The total knowledge score for each participant was calculated by summing item scores and converting them into a percentage of the maximum possible score using the following formula:

(Obtained score ÷ Maximum possible score) × 100.

Based on author consensus, scores were classified as low (<33%), average (33%-66%), and adequate (>66%) knowledge. These thresholds were chosen to reflect practical benchmarks for understanding, where <33% was interpreted as minimal understanding, and >66% as sufficient for clinical application. These categories were predefined prior to data analysis and were used for descriptive stratification in this exploratory study. These cutoffs were not intended to imply clinical competency standards but to allow relative comparison across participants.

## Results

Demography

A total of 135 postgraduate dental students pursuing their Master of Dental Surgery (MDS) qualification in Paediatric and Preventive Dentistry responded to the questionnaire. As recruitment occurred through multiple messaging platforms and institutional forwarding, the exact number of recipients could not be determined; therefore, a response rate could not be calculated. No formal non-response analysis was feasible due to the anonymous distribution method. A summary of participant characteristics is presented in Table 1. Of the respondents, 31 (23%) were from government-run institutions, while the remaining 104 (77%) were from private institutions. Regarding their year of study, 33.3% (45 individuals) were in their first year, 48.8% (49 individuals) were in their second year, and 17.7% (41 individuals) were in their third year. Geographically, 41.5% (56 individuals) were from North India, 8.9% (12 individuals) from East India, 12.6% (17 individuals) from West India, and 37.0% (50 individuals) from South India.

**Table 1:** Sociodemographic characteristics of the study participants (N=135)

Variable	Category	Frequency (number)	Percentage (%)
Year of study	First year	45	33.3
	Second year	49	36.3
	Third year	41	30.4
Type of institution	Government	31	22.9
	Private	104	77.1
Geographic region	North India	56	41.5
	South India	50	37.0
	East India	12	8.9
	West India	17	12.6

Knowledge

The test-retest reliability analysis results revealed a kappa value of 0.96, indicating an “almost perfect agreement.” The data were analyzed, and the knowledge levels of the respondents were quantified as percentages. Based on author consensus, a score of less than 33% was categorized as low knowledge, a score between 33% and 66% was considered average knowledge, and a score exceeding 66% indicated adequate knowledge of the subject in question. Among the 135 participants, 25.9% (35 individuals) scored below 33% in the knowledge assessment, while 62.2% (84 individuals) scored between 33% and 66%. Only 11.8% (16 individuals) scored above 66%. The average knowledge level was calculated to be 46.4%, with a standard deviation of 12.5%, ranging from 5.26% to 78.95%. The respondents showed good knowledge regarding the diagnosis, patient selection, and appliance fabrication, while more technical aspects, such as cephalometric analysis and bite registration, yielded mixed responses (Table 2).

**Table 2:** Knowledge level of the participants SD: standard deviation

Knowledge level	Number (N=135)	%
<33% (low)	35	25.9%
33%-66% (average)	84	62.2%
>66% (adequate)	16	11.8%
Mean±SD (range) (%)	46.4±12.5 (5.26-78.95) (%)	

Regarding the year of study, second- and third-year students exhibited higher knowledge levels compared to first-year students. Among first-year students, 46.6% (21 individuals) had knowledge levels below 33%, 44.4% (20 individuals) were within the average range, and 8.8% (four individuals) demonstrated adequate knowledge. In contrast, 69.3% (34 individuals) of second-year students and 73.1% (30 individuals) of third-year students displayed average knowledge of the topic. This difference is expected, as knowledge generally increases with academic progression. The Chi-square test revealed a significant difference in knowledge levels across different years of study (χ²=15.28, p=0.004). A higher percentage of respondents with an adequate knowledge level of TBFT were employed in government settings compared to private institutes, and this difference was statistically significant (χ²=7.31, p=0.025). However, there was no statistically significant difference observed in the geographical distribution of respondents, with a higher percentage consistently falling within the average knowledge level category across all four regions of the country (Table 3).

**Table 3:** Distribution of knowledge level of the participants based on year pursuing, workplace type, and geographical region

Variable		Knowledge level				Significance
		Total number	<33% (low)	33%-66% (average)	>66% (adequate)	
Year pursuing	First year	45	46.6%	44.4%	8.8%	Chi-square=15.28, p=0.004
	Second year	49	16.3%	69.3%	14.2%	
	Third year	41	14.6%	73.1%	12.1%	
Workplace	Government	31	3.2%	77.4%	19.6%	Chi-square=7.31, p=0.025
	Private	104	14.4%	80.8%	4.8%	
Region	North India	56	16.1%	78.6%	5.4%	Chi-square=2.68, p=0.847
	East India	12	8.3%	83.3%	8.3%	
	West India	17	5.9%	82.4%	11.8%	
	South India	50	10.0%	80.0%	10.0%	

Attitude

Among the 135 participants, our analysis revealed that 78.5% (106 individuals) had a positive attitude toward the success and treatment potential of TBFT, and 17.0% (23 individuals) had a strongly positive attitude. Additionally, 48.9% (66 individuals) of the respondents considered TBFT to be effective in treating Angle’s Class II malocclusion, while 23.7% (32 individuals) believed it to be successful but with unpredictable outcomes. When asked about the incidence of relapse, nearly 86% (116 individuals) of respondents reported a moderate to low incidence of relapse, whereas 10% (14 individuals) considered the incidence to be extremely low. Patient compliance also significantly influences the outcome of TBFT; 88.2% (119 individuals) of the respondents reported moderate to low compliance, while only 8.1% (11 individuals) reported high compliance. Overall, the participants showed a positive attitude toward TB therapy, emphasizing that patient compliance is crucial for successful treatment outcomes.

Practice

Among the 135 respondents, 70.4% (71 individuals) had never treated a patient with Angle’s Class II malocclusion, including all first-year students and a majority of second-year students, while 29.6% (64 individuals), comprising only second- and third-year students, reported having done so. Among those who had treated such cases, the majority (79.7% or 51 individuals) preferred using removable myofunctional appliances, most commonly the Twin Block, which was the focus of this study. A significant majority (74.8% or 101 individuals) believed that the outcome of treatment depends on the patient’s developmental age, with 87.4% (118 individuals) favoring treatment during the mixed dentition stage. Regarding appliance use during meals, 43% (58 individuals) advised their patients to remove the appliances, whereas 57% (77 individuals) recommended keeping them in place while eating. Additionally, nearly 42% (57 individuals) of respondents indicated that using labial bows improved the treatment outcome by correcting incisor tipping (Table 4).

**Table 4:** Distribution of the participants based on experience in treating Class II malocclusion

Treatment status			Number (N=135)	%
Ever treated a patient with Angle’s Class II malocclusion	No	First year	45	33.3
		Second year	22	16.3
		Third year	04	3.0
	Yes	First year	0	0.0
		Second year	23	17.0
		Third year	41	30.4
If yes, then the technique	Camouflaging by performing premolar extractions		7	10.9
	Removable myofunctional appliances		51	79.7
	Fixed myofunctional appliance		6	9.4

While statistical significance was observed across certain subgroups, effect size measures were not calculated, and therefore, the practical magnitude of these differences should be interpreted cautiously.

## Discussion

In orthodontics, developing Class II malocclusions are among the most commonly encountered cases. In children with Class II malocclusion, identifiable signs often appear early on, even before the permanent teeth fully emerge. Once this malocclusion pattern sets in, it tends to persist throughout development, with minimal ability for self-correction as the child grows [11,12]. The Twin Block appliance has become the leading and most widely used functional device for treating Class II malocclusion in young patients [13,14].

Baccetti et al. [15] suggest that functional appliance therapies achieve maximum therapeutic effects when timed with the pubertal mandibular growth spurt, particularly around Cervical Vertebral Maturation Index (CVMI) stage 3 or the fourth and fifth epiphyseal stages, as described by Björk [16]. This age group comes within the prerogative of a pediatric dentist and makes it imperative that they are well-versed in treating children with Class II malocclusion using this technique. Thus, our study aimed to evaluate the overall status of awareness among the postgraduate students of pediatric dentistry, along with their perception and level of clinical experience with the TB myofunctional appliance. Several studies affirm that the TB appliance is effective for Class II correction by promoting mandibular advancement and enhancing skeletal and facial aesthetics [17-20]. Beyond skeletal correction, systematic evidence has demonstrated that functional appliances can also produce measurable improvements in external soft tissue profile, facial convexity, and aesthetic parameters during adolescence, further reinforcing their clinical significance in comprehensive dentofacial management [8].

The respondents showed a good level of knowledge regarding the treatment under study, although the overall knowledge score was in the lower range of average. The respondents demonstrated a strong understanding of diagnosis, patient selection, and appliance fabrication. However, responses varied regarding more technical aspects of treatment, such as cephalometric analysis and bite registration, exposing the rift between knowledge and clinical experience or practice, as more than half of the respondents in this study have never treated a patient with Angle’s Class II malocclusion.

Although Clark recommends full-time TB appliance wear [21], recent studies [22] suggest similar efficacy with part-time use, favoring patient comfort. However, many respondents in our study (43% or 58 individuals) reported advising removal during meals, likely for better compliance.

Patient compliance with TBFT is critical for its success in treating Class II malocclusion, yet it remains a significant challenge. Studies indicate that adherence to the prescribed wear schedule is often suboptimal. For instance, Chandorikar et al. highlighted that discomfort and lifestyle disruptions are primary factors leading to non-compliance, proposing a semi-fixed TB design to address these issues and ensure better adherence [22]. Additionally, Al-Moghrabi et al. found that many patients struggle with consistent full-time wear, which can hinder treatment effectiveness and also frequently overestimate wear durations during follow-up visits [23]. Their systematic review outlines the need for continuous patient motivation and regular follow-up visits to monitor and encourage adherence. In our present study, an overwhelming majority (88.2% or 119 individuals) reported moderate to low compliance with treatment in their clinical practice. Overall, while the TB appliance is effective, overcoming compliance challenges through innovative designs, patient education, and supportive follow-up care is essential for achieving optimal treatment outcomes.

Nearly 78% (105 individuals) of the respondents in this study had a positive attitude toward performing TBFT and its potential in effectively correcting Angle’s Class II malocclusions. However, this attitude failed to translate entirely into practice, as evidenced in our current study.

The findings suggest that within the scope of current training, there may be opportunities to enhance clinical exposure and structured teaching related to TB therapy. The postgraduate MDS curriculum of Paediatric and Preventive Dentistry lists “Myofunctional therapy” as one of the core components of the program. However, the specific learning objectives within the topic are not defined. A broad topic such as “Myofunctional therapy” can be interpreted in different and diverse ways by the educators delivering the curriculum in the absence of a structured competency framework. Such diversity also leads to heterogeneity in learning outcomes across different institutions. The authors suggest that the absence of a structured competency framework for such critical topics in the postgraduate curriculum may correlate with lower knowledge and practice scores. Internationally, postgraduate orthodontic curricula, such as those in the UK and Australia, outline structured competencies for functional appliance training, including case-based learning, mandatory logbooks, and supervised delivery of removable appliances such as the Twin Block. Indian postgraduate programs could consider adapting similar outcome-based strategies, incorporating structured clinical postings, simulation-based workshops, and direct mentorship models. Such enhancements would align training with global standards and ensure students gain both theoretical and hands-on proficiency in managing skeletal Class II malocclusion with functional appliances. Our findings may encourage educators to strengthen clinical training in TB therapy. A suggestive framework of “Myofunctional therapy” is demonstrated in Table 5 as an example.

**Table 5:** Suggestive competency framework of “Myofunctional therapy” for postgraduate students of pediatric dentistry

Competency	Level expected
Diagnose skeletal malocclusions in growing children requiring myofunctional therapy based on clinical and radiographic features	Should know/should know how to/should show how to/should be able to do
Identify the need and rationale for interception of skeletal malocclusion in growing children using myofunctional therapy	Should know/should know how to/should show how to
Describe different myofunctional appliances for interceptive orthodontics in the pediatric population (design, indications, contraindications, advantages, and disadvantages)	Should know/should know how to
Develop a treatment plan using myofunctional appliances for intercepting skeletal malocclusion of a growing child based on clinical and radiographic findings	Should know/should know how to/should show how to/should be able to do
Intercept skeletal malocclusions by delivering a myofunctional appliance to a growing child	Should know/should know how to/should show how to/should be able to do
Resolve challenges in myofunctional therapy delivered to a growing child to intercept skeletal malocclusion	Should know/should know how to/should show how to/should be able to do

This table identifies key skills postgraduate students should acquire, categorized by expected levels of proficiency based on Miller’s pyramid. By defining competencies explicitly, the framework aims to bridge the gap between theoretical understanding and clinical application, guiding educators in curriculum planning and helping standardize training across institutions.

While this study did not directly assess barriers faced by postgraduate students, insights from the focus group discussions and open-ended survey responses suggested common challenges in the clinical application of Twin Block therapy. These included limited case exposure, ambiguity regarding treatment timing and appliance design, and variability in departmental protocols. Addressing such challenges through structured academic reinforcement and clinical demonstrations may enhance both confidence and competence. The suggestions outlined in Table 5 were developed in light of these findings and aim to serve as a guiding framework for future academic enrichment.

While the suggested framework may align with expected learning outcomes, it is important to note that no dedicated curriculum or standardized competency structure currently exists in most academic institutions specifically for Twin Block therapy. The proposed milestones are meant to serve as a foundational guide for structuring future curriculum components, rather than implying current deficiencies. These exploratory recommendations aim to support expert panels in formalizing consistent postgraduate competencies across teaching institutions.

This study has several limitations. The use of convenience sampling through professional networks may have introduced selection bias, as respondents with a greater interest in orthodontic practice may have been more likely to participate. A formal sample size calculation was not performed due to the exploratory design, which may limit statistical generalizability. Additionally, findings are based on self-reported data and are therefore subject to recall bias and social desirability bias, particularly in reporting clinical practices and compliance perceptions. Although the survey was anonymous and did not collect personal identifiers, the absence of formal institutional ethical approval may be viewed as a methodological limitation. Accordingly, the findings should be interpreted as preliminary and hypothesis-generating rather than definitive.

The authors realize that including first-year postgraduate students in the study might have impacted the overall results since they have less knowledge and clinical exposure when compared to their academic seniors. However, we believe that involving them in the study was necessary to fully comprehend the continuum of knowledge and practice as the students move up the academic ladder. Besides, the study was conducted toward the mid to end of the academic year, ensuring that no freshers, who were completely unexposed to the treatment modality, were included in this study.

## Conclusions

This exploratory study provides preliminary insights into the knowledge, attitude, and practice patterns related to Twin Block therapy among pediatric dentistry postgraduates in India. Although attitudes toward TBFT were generally positive, fewer respondents demonstrated adequate knowledge or reported direct clinical experience. As a cross-sectional survey, the study cannot determine the underlying reasons for this observed gap. However, the findings suggest that structured clinical exposure and clearly defined competency frameworks may help strengthen training in functional appliance therapy. These recommendations should be interpreted as educational considerations rather than definitive curricular prescriptions.

## Appendices

Participant consent

1. I agree to participate in the research study. I understand the purpose and nature of this study, and I am participating voluntarily. I understand that I can withdraw from this study at any time without any penalty or consequences.

Mark only one oval.

I agree

I disagree

2. Please provide your email ID.*

3. Which of the following best describes you?*

Mark only one oval.

Pursuing MDS in Paediatric Dentistry

Completed MDS in Paediatric Dentistry

Other:

4. If you are pursuing MDS in Paediatric Dentistry, then which year do you belong to?

Mark only one oval.

First year

Second year

Third year

5. Which of the following best describes your workplace?*

Mark only one oval.

Government Dental College and Hospital

Private Dental College and Hospital

Independent Dental Practitioner

6. Which part of India do you belong to?*

Mark only one oval.

North India

South India

East and North-East India

West India

Twin Block functional therapy survey

7. Have you ever treated a patient with Angle’s Class II malocclusion in your practice?*

Mark only one oval.

Yes

No

8. If you responded “Yes” to the previous question, then which techniques have you used to treat such patients? (You may choose more than one answer.)

Check all that apply.

Removable myofunctional appliances, such as Twin Block

Fixed myofunctional appliance

Camouflaging by using Class II elastics

Camouflaging by performing premolar extractions

Other:

9. The main aim of Twin Block therapy in Class II division 1 malocclusion is to improve the patient profile (positive VTO):

Mark only one oval.

Strongly agree

Agree

Neutral

Disagree

Strongly disagree

10. Non-improvement of profile on mandibular advancement is an absolute contraindication for Twin Block therapy.

Mark only one oval.

Strongly agree

Agree

Neutral

Disagree

Strongly disagree

11. During which stage of development would you prefer to do Twin Block functional therapy? 

Mark only one oval.

Mixed dentition

Transitional dentition

Permanent dentition

Twin block therapy can be performed at any stage of development

12. According to Clarke, which of the following three angles determine basic pattern of facial growth?

Mark only one oval.

Craniomandibular angle, facial axis angle, mandibular plane angle

Facial plane angle, cranial base angle, mandibular plane angle

Facial plane angle, facial axis angle, cranial base angle

Facial axis angle, mandibular plane angle, cranial base angle

13. Which is your preferred angulation for bite blocks during Twin Block therapy? 

Mark only one oval.

45 degrees

60 degrees

70 degrees

90 degrees

14. How much interincisal clearance do you prefer to keep during bite registration for Twin Block functional therapy?

Mark only one oval.

2 mm

3 mm

4 mm

6 mm

15. During fabrication of the Twin Block appliance, the bite blocks are constructed to be a few mm less than the freeway space.

Mark only one oval.

Strongly agree

Agree

Neutral

Disagree

Strongly disagree

16. Labial bow is not a necessary component of the Twin Block appliance and may actually worsen the final outcome of treatment.

Mark only one oval.

Strongly agree

Agree

Neutral

Disagree

Strongly disagree

17. Do you prefer to use a pre-activated labial bow in your appliance to reduce overjet at the beginning of the treatment?

Mark only one oval.

Yes (Reducing overjet by incisal tipping improved treatment outcome)

No (Reducing overjet by incisal tipping worsened treatment outcome)

18. Do you think that lower incisor capping is mandatory in treating Class II division 1 malocclusion cases with the Twin Block technique?

Mark only one oval.

Yes

No

19. The main aim of the active phase of the treatment in the Twin Block functional therapy is to correct overjet, overbite, and disto-occlusion.

Mark only one oval.

Strongly agree

Agree

Neutral

Disagree

Strongly disagree

20. Trimming of the upper block is done occluso-distally.

Mark only one oval.

Strongly agree

Agree

Neutral

Disagree

Strongly disagree

21. The Twin Block appliance should be removed while eating.

Mark only one oval.

Strongly agree

Agree

Neutral

Disagree

Strongly disagree

22. How successful have you found this technique in improving aesthetics in Angle’s Class II malocclusion patients?

Mark only one oval.

Very successful >90%

Successful 70%-90%

Moderately successful 50%-70%

Unsuccessful

Very unsuccessful

23. What is your experience regarding patient compliance while wearing the Twin Block appliance?

Mark only one oval.

Very high compliance >90%

High compliance 60%-90%

Moderate compliance 30%-60%

Low compliance 10%-30%

Very low compliance >10%

24. What is your experience regarding relapse of malocclusion after the Twin Block functional therapy?

Mark only one oval.

Very high incidence >90%

High incidence 60%-90%

Moderate incidence 30%-60%

Low incidence 10%-30%

Very low incidence >10%

25. From your clinical experience, do you think that the final outcome of the treatment is dependent upon the developmental stage of the patient (independent of treatment duration)?

Mark only one oval.

Yes (Treatment outcome is better in younger patients than in adults.)

No (Treatment outcome remains the same for both younger and adult patients.)

Other:
